# Alleviating Depressive Symptoms Through Continuous Positive Airway Pressure Therapy: Long-Term Effects on Patients with Obstructive Sleep Apnea

**DOI:** 10.1055/s-0046-1819597

**Published:** 2026-11-06

**Authors:** Yutaka Kozu, Hisato Hiranuma, Yasunori Itoda, Yusuke Jinno, Shun Yokota, Shuichiro Maruoka, Yasuhiro Gon

**Affiliations:** 1 Division of Respiratory Medicine, Department of Internal Medicine, Nihon University School of Medicine, TokyoJapan

**Keywords:** depression, continuous positive airway pressure, sleep, sleep apnea

## Abstract

**Background:**

Obstructive sleep apnea (OSA) increases the risk of physical and psychological comorbidities, including depression. Although the short-term impact of continuous positive airway pressure (CPAP) therapy on depressive symptoms is established, its long-term psychological impact beyond 2 years remains underexplored.

**Objective:**

To evaluate the impact of long-term CPAP therapy on depressive symptoms in patients with OSA, focusing on Self-Rating Depression Scale (SDS) subdomain changes, as well as the influence of demographic and clinical variables such as age and sex.

**Materials and Methods:**

We enrolled 170 patients with OSA who underwent polysomnography and received CPAP for ≥ 6 months at the Nihon University Itabashi Hospital Sleep Center. Depression severity was measured using the SDS, and subgroup analyses assessed associations between changes in SDS scores and clinical parameters (age, sex, CPAP adherence, and sleep-related indices).

**Results:**

Total SDS scores significantly declined from 40.2 to 37.5 in patients using CPAP for ≥2 years. Improvement was most pronounced in the cognitive and behavioral subdomains (e.g., fatigue, motivation, and decision-making) and was statistically significant among male patients, whereas female patients showed a nonsignificant trend toward improvement. No significant associations were observed between baseline characteristics (e.g., age and comorbidities) and SDS score changes. Multivariate analysis confirmed these improvements were not solely attributable to a single subdomain.

**Conclusion:**

Long-term CPAP therapy was associated with modest improvements in depressive symptoms in patients with OSA, particularly in cognitive and behavioral domains. In exploratory analyses, benefits appeared more evident in males, but these findings should be interpreted with caution given the limited female sample size.

## Introduction

Obstructive sleep apnea (OSA) is a common sleep-related breathing disorder characterized by recurrent episodes of partial or complete upper airway obstruction during sleep, leading to intermittent hypoxia, sleep fragmentation, and excessive daytime sleepiness. Although OSA affects a substantial proportion of the adult population, particularly middle-aged and older individuals, it remains underdiagnosed and undertreated in many cases. 1 Untreated OSA is associated with various adverse health outcomes, including hypertension, cardiovascular disease, metabolic syndrome, and neurocognitive impairment. The high burden of comorbidities underscores the importance of early diagnosis and comprehensive management of this condition. 2 3

In addition to its physical consequences, OSA has significant psychological implications. A growing body of evidence suggests a strong association between it and depressive symptoms. 4 5 Patients with OSA are more likely to suffer from depression compared to the general population, with reported prevalence rates ranging from 20 to 60%, depending on the population studied and the criteria used. 6 7 Moreover, this relationship appears bidirectional: depression can exacerbate sleep disturbances, while untreated OSA may intensify mood disorders. 8 Moreover, comorbid insomnia and OSA have been shown to synergistically increase the severity of depressive symptoms. 9 Although some studies have questioned the causality of this association, more recent investigations have supported a robust link. 10 11

Continuous positive airway pressure (CPAP) is the first-line therapy for moderate-to-severe OSA and has been proven beneficial in improving daytime alertness, sleep quality, and cardiovascular health. 12 Although the efficacy of CPAP in alleviating physical symptoms is well established, its impact on mood and mental health remains unclear. Several studies have reported a reduction in depressive symptoms following CPAP therapy, particularly in patients with preexisting depression or excessive daytime sleepiness. 13 14 However, the extent of improvement varies widely, and not all studies have shown statistically significant changes in depression scores. For example, previous studies reported no meaningful improvement after 3 months, 15 16 while others, including Pattison et al., 17 demonstrated lasting emotional and cognitive improvements over 12 months. 18 19

The importance of adherence has been emphasized, with more consistent CPAP use being associated with greater improvements in mood. A recent study using the Hospital Anxiety and Depression Scale (HADS) revealed a modest but statistically significant decrease in depression scores among adherent users (Cohen's *d* = 0.18). 20 However, these findings are limited to short- or mid-term follow-up (3–12 months), and little is known about outcomes beyond this timeframe.

The potential of long-term CPAP therapy in alleviating depressive symptoms remains unclear. Although previous reports have indicated improvements in depressive symptoms within the first few months of treatment, there is a notable lack of studies investigating psychological outcomes in patients who have used CPAP for ≥2 years. In a large-scale cohort study (the SAVE trial), long-term treatment (>3.7 years) was associated with a reduced incidence of depression, although symptom-specific analyses were limited. 21 Similarly, improvements in depression were observed in a 3-year follow-up study that compared CPAP users and non-users. 22 However, few studies have examined the changes in specific domains of depressive symptoms, such as mood, cognition, and motivation, over extended periods.

Furthermore, although previous studies have frequently used instruments, including the 9-item Patient Health Questionnaire or HADS, little is known about how CPAP adherence affects outcomes measured using the Self-Rating Depression Scale (SDS), a multidimensional tool that assesses depressive mood, physical symptoms, cognitive and behavioral changes, and functional impairments. One study indicated an association between CPAP use and improvements in SDS scores. However, detailed analyses of subscale changes and long-term adherence are lacking. 23

Therefore, this study aimed to examine the long-term impact of CPAP therapy on depressive symptoms in patients with OSA, focusing on changes in SDS scores and their subdomains. We analyzed data from a cohort of patients who had used CPAP for ≥2 years and evaluated changes in total SDS and component (cognitive, behavioral, and functional domains) scores. To our knowledge, this is the first study to investigate detailed SDS-based changes over a long treatment duration. By clarifying the psychological effects of sustained CPAP use, this study sought to advance a more holistic understanding of OSA management and promote better adherence by recognizing its mental health benefits.

## Materials and Methods

### Study Design

This study was a single-center observational pre–post (within-subjects) study designed to examine the effects of long-term CPAP therapy on depressive symptoms in patients with OSA. We evaluated changes in SDS scores before and after the initiation of CPAP therapy using routinely collected clinical data over a treatment period exceeding 6 months.

### Participants and Sample Size Justification

Participants were recruited from the Nihon University's Itabashi Hospital Sleep Center. Eligible participants were adults diagnosed with OSA who underwent full-night polysomnography and received CPAP therapy for at least 6 months. A total of 170 patients (mean age 53 ± 15 years, 82.9% male) who provided informed consent and completed baseline and follow-up SDS assessments were included.

A post hoc sample size estimation was conducted using the observed effect size (Cohen's *d* = 0.25) in SDS score reduction, with a two-tailed α = 0.05 and power (1 − β) = 0.80. The required sample size was estimated to be 126 participants. Thus, a sample size of 170 patients was sufficient to detect clinically meaningful changes. This post hoc calculation targeted the primary within-subject pre–post change in total SDS and was not designed to detect sex-by-time interactions; accordingly, sex-stratified analyses were exploratory and underpowered.

In this cohort, approximately half the participants demonstrated adherence by the conventional threshold (≥ 4 h/night on ≥ 70% of nights), which allowed for balanced group comparisons in exploratory subgroup analyses.

A total of 200 patients with OSA initiating CPAP were screened. Among those, 30 patients were excluded due to ineligibility or declined participation. Finally, 170 patients were enrolled. Among them, SDS data were available for 164 at baseline and 166 at follow-up. As such, 160 patients had complete paired data and were included in the primary pre–post analysis. Subgroup analyses were conducted according to CPAP duration (< 2 years, n = 66; ≥2 years, n = 104) and in a subset with complete covariates (n = 140), as shown in Fig. 1 .

**Fig. 1:**
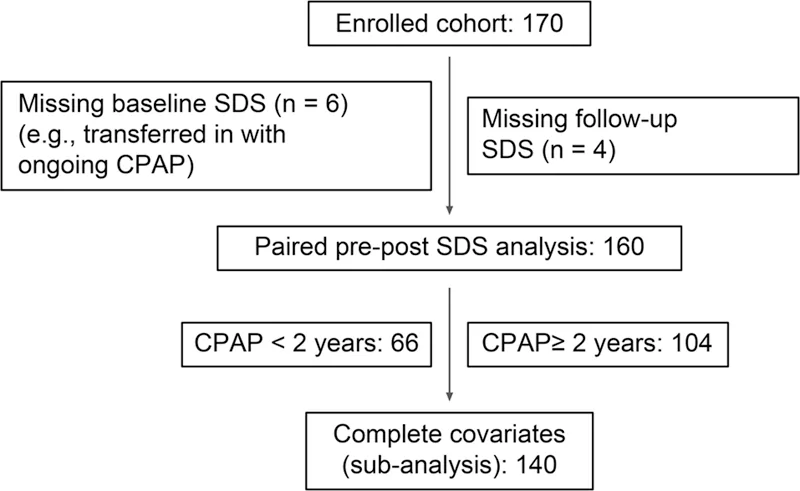
Participant flow and population analysis of 200 patients screened, 170 were enrolled. Baseline SDS data were available for 164 participants and follow-up data for 166; 160 participants with paired SDS measurements were included in the primary analysis. Subgroup analyses were conducted by CPAP duration (< 2 years, n = 66; ≥ 2 years, n = 104) and for a subset with complete covariates (n = 140).

### Data Collection Procedures

All participants underwent polysomnography as part of their OSA diagnostic assessment.

Adherence data regarding CPAP therapy were obtained from the manufacturer's online cloud services. Indicators included use ≥ 4 h/night on ≥ 70% of nights, mean nightly usage, and residual apnea–hypopnea index (AHI).

Depressive symptoms were assessed using the SDS, a questionnaire designed to measure the severity of depressive states. The scores were collected at baseline (before CPAP therapy) and at the end of the study period. The symptoms were defined based on raw scores: 40 to 47 for mild, 48 to 55 for moderate, and ≥ 56 for severe. The validated Japanese paper-based version of the SDS was used and was self-administered under supervision. 24

### Statistical Analysis

Patients were grouped based on CPAP usage duration (< 2 vs. ≥ 2 years) guided by clinical relevance and precedent in previous research. Long-term use of CPAP, particularly beyond the 2-year mark, has been associated with greater physiological and psychological stabilization. This threshold has been commonly used to assess sustained effects on sleep architecture and mental health.

Data were analyzed using appropriate statistical techniques to assess changes in the SDS scores from pre- to post-CPAP therapy. The primary endpoint was the difference in total SDS scores before and after treatment. Welch's t-test and two-way analysis of variance were used to compare the means between groups. For the primary within–subject pre–post comparison of total SDS, paired t–tests were used or, when assumptions were not met, Wilcoxon signed–rank tests. Between–group comparisons (< 2 vs. ≥ 2 years) employed t–tests or Mann–Whitney U tests as appropriate. The two–sided α value was 0.05. Cluster analysis (Ward's method) and Tukey's honest significant difference test were used for subgroup analyses.

All statistical analyses were primarily performed using the JMP Pro (SAS Institute) software, version 17.0, and additional analyses were conducted using the IBM SPSS Statistics (IBM Corp.), version 26. To contextualize clinical relevance without additional analyses, we interpreted the magnitude of change against widely cited distribution–based minimal clinically important difference (MCID) benchmarks for patient–reported outcomes (standard deviations [SD] ≈ 0.3–0.5). Standardized change was summarized as the mean pre–post difference divided by the baseline SD; no anchor–based MCID or responder analyses were performed.

### Analytical Approach

In line with the redefined analysis plan and based on the preliminary dataset and presentation materials, the following approach was adopted. A comprehensive analysis was conducted using data from all 170 study participants. To identify the clinical subgroups of CPAP-treated patients likely to show improvements in depressive symptoms, we performed univariate and multivariate analyses using the SDS scores pre- and posttreatment, as well as score changes as dependent variables. The multivariate analyses included cluster analysis, multiple linear regression, and multivariate logistic regression to examine associations between clinical characteristics and changes in SDS scores.

### Ethical Considerations

The present study was approved by the Ethics Committee of the Nihon University's Itabashi Hospital (Approval Number: RK-1705097). All participants provided informed consent before inclusion in the study, and all procedures were conducted in accordance with the Declaration of Helsinki and relevant guidelines.

## Results

### Participant Demographics and Baseline Characteristics

The study encompassed 170 patients diagnosed with OSA undergoing CPAP therapy. A total of 141 males and 29 females, with a mean age of 53 ± 15 years, were included. Overall, 16 (9.4%) participants had depression ( Table 1 ).

**Table 1:** Clinical characteristics of participants (n = 170)

Variable	Value
Male, n (%)	141 (82.9%)
Age, years (mean ± SD)	53 ± 15
BMI, kg/m ^2^ (mean ± SD)	28 ± 5.9
Depression, n (%)	16 (9.4%)
Hypertension, n (%)	76 (44.7%)
Diabetes mellitus, n (%)	33 (19.4%)
Chronic heart failure, n (%)	19 (11.2%)
Ischemic heart disease, n (%)	11 (6.5%)
Atrial fibrillation, n (%)	12 (7.1%)
Arrhythmia (excluding AF), n (%)	2 (1.2%)
Dyslipidemia, n (%)	28 (16.5%)
Hyperuricemia, n (%)	12 (7.1%)
Cerebrovascular disease, n (%)	6 (3.5%)
Malignancy, n (%)	8 (4.7%)
Nasopharyngeal disorders, n (%)	7 (4.1%)
Schizophrenia or psychiatric disorders, n (%)	2 (1.2%)
Collagen vascular disease, n (%)	3 (1.8%)
Interstitial lung disease, n (%)	3 (1.8%)

**Abbreviations:** AF, atrial fibrillation; BMI, body mass index; SD, standard deviation.

Polysomnography data were divided based on the duration of CPAP therapy (< 2 and ≥ 2 years). Total in-bed time averaged approximately 543 min, with no significant difference between the groups. Total sleep time was significantly longer in the group with ≥ 2 (444.84 min) than in that with < 2 (399.22 min) years of therapy. At baseline, participants who subsequently used CPAP for ≥ 2 years had longer total sleep time and higher sleep efficiency than those with < 2 years; the arousal index, number of awakenings, and limb movement indices were also lower in those using CPAP for ≥ 2 years. Rapid eye movement (REM) sleep latency and the percentages of different sleep stages (WK, N1, N2, N3, and REM) exhibited no significant differences between the groups.

The mean and lowest SpO _2_ levels, along with the 3% oxygen desaturation index, demonstrated no significant differences between the groups. The apnea, hypopnea, and AHI scores indicated significant improvements in patients with a longer duration of CPAP therapy ( Table 2 ).

**Table 2:** Polysomnography parameters by CPAP usage duration

Parameter	Overall Mean ± SD	< 2 Years Mean ± SD	≥ 2 Years Mean ± SD	*p* -value
Total time in bed, min	542.50 ± 27.95	538.35 ± 27.90	545.09 ± 27.82	0.099
Sleep period time, min	519.19 ± 46.58	513.25 ± 44.54	522.83 ± 47.66	0.076
Total sleep time, min	427.72 ± 74.22	396.08 ± 83.26	447.45 ± 60.51	< 0.001***
Wake after sleep onset, min	92.04 ± 61.89	118.91 ± 73.22	74.53 ± 45.79	< 0.001***
Sleep efficiency, %	78.85 ± 12.89	73.88 ± 14.96	81.98 ± 10.30	0.001**
Sleep latency, min	17.28 ± 31.45	16.41 ± 29.17	17.83 ± 32.97	0.678
REM sleep latency, min	170.86 ± 100.81	164.75 ± 99.01	174.72 ± 102.31	0.593
Stage WK, %	17.82 ± 12.12	23.57 ± 14.40	14.28 ± 8.86	< 0.001***
Stage N1, %	51.13 ± 37.57	47.87 ± 17.73	53.21 ± 45.94	0.583
Stage N2, %	34.45 ± 14.08	33.62 ± 14.06	34.97 ± 14.14	0.527
Stage N3, %	4.27 ± 5.71	5.22 ± 6.15	3.67 ± 5.35	0.047*
Stage REM, %	13.69 ± 11.64	14.15 ± 10.08	13.40 ± 12.58	0.339
Arousal index (/h)	59.19 ± 22.04	58.45 ± 18.37	59.66 ± 24.18	0.991
Number of arousals	423.42 ± 173.58	378.32 ± 108.80	451.48 ± 199.24	0.032*
Limb movement index (/h)	14.82 ± 19.76	20.53 ± 23.50	11.25 ± 16.15	0.006**
Periodic limb movement index (/h)	10.24 ± 17.18	14.30 ± 20.31	7.70 ± 14.45	0.006**
Mean SpO _2_ (%)	94.63 ± 2.84	94.91 ± 2.39	94.46 ± 3.08	0.704
Lowest SpO _2_ (%)	76.14 ± 14.19	79.53 ± 11.15	74.02 ± 15.49	0.031*
3% oxygen desaturation index (/h)	60.23 ± 28.4	86.72 ± 30.0	43.65 ± 29.62	0.139
Apnea index (/h)	23.75 ± 24.69	16.34 ± 16.77	28.39 ± 27.64	0.032*
Mean apnea duration (s)	23.92 ± 13.80	22.02 ± 7.87	25.09 ± 16.35	0.160
Hypopnea index (/h)	24.69 ± 16.37	28.04 ± 16.20	22.59 ± 16.21	0.052
Mean hypopnea duration (s)	24.41 ± 6.05	24.93 ± 5.55	24.10 ± 6.34	0.438
Apnea–hypopnea index (/h)	48.44 ± 25.63	44.38 ± 22.17	50.98 ± 27.39	0.110
Number of apneas and hypopneas	345.32 ± 204.13	279.88 ± 138.74	385.59 ± 227.01	0.001**
Total duration of apneas and hypopneas (min)	150.73 ± 102.37	117.54 ± 65.18	171.38 ± 115.44	0.001**
Snoring (% of total sleep)	34.21 ± 26.35	25.05 ± 25.21	39.67 ± 25.61	0.001**

**Abbreviations:** REM, rapid eye movement; SD, standard deviation. **Notes:** Values are expressed as mean ± standard deviation. Statistical comparisons between groups were performed using the Mann–Whitney U test. The *p-* values had significance levels of *<0.05,**<0.01, ***< 0.001.

The polysomnography data in Table 2 were collected before CPAP therapy initiation. These baseline sleep characteristics—longer total sleep time and higher sleep efficiency—may be linked to improved long-term treatment adherence. This suggests that specific pretreatment sleep profiles might predict sustained compliance with therapy.

### Therapy Adherence

The average duration of CPAP use among the participants was 920 ± 506 days (median: 876 days), corresponding to approximately 2.5 years. Accordingly, we categorized participants into two groups: < 2 (n = 66, 38.8%) and ≥ 2 (n = 104; 61.2%) years of CPAP use. The overall average daily usage time was 281 ± 134 min, and the percentage of nights with CPAP use exceeding 4 h was 62.8 ± 33.8%. The mean residual AHI was 3.3 ± 4.2 events/h.

Among participants who had used CPAP for < 2 years, the average daily usage time was 255 ± 136 min, the percentage of nights with CPAP use exceeding 4 h was 57.5 ± 35.6%, and the mean residual AHI was 2.8 ± 3.1 events/h.

Among those who used CPAP for ≥ 2 years, the average daily usage time was 296 ± 130 min, the percentage of nights with CPAP use exceeding 4 h was 64.8 ± 33.8%, and the mean residual AHI was 3.6 ± 4.7 events/h.

Adherence metrics were used to evaluate the relationship between CPAP use and changes in depressive symptoms.

### Changes in Depressive Symptoms

After long-term CPAP therapy, a significant reduction in depressive symptoms was observed among the participants. The total SDS scores decreased from an average of 38.9 before to 36.8 after therapy, and this change was statistically significant ( *p* = 0.02). For participants using CPAP for < 2 years, SDS scores decreased from 38.2 to 36.3; however, this was not statistically significant ( *p* = 0.2038). Participants with CPAP usage of ≥ 2 years showed a significant reduction in SDS scores from 40.2 to 37.5 ( *p* = 0.0474).

In exploratory sex–stratified analyses ( Supplementary Table S1 ), males showed a significant reduction in total SDS, whereas females exhibited a directionally similar but nonsignificant change; given the small female sample and multiple comparisons, these results should be interpreted cautiously. In exploratory analyses, male participants experienced a reduction in SDS scores from 38.3 to 35.4 ( *p* = 0.0490). Female participants showed a decrease from 42.4 to 38.5, although this was not statistically significant ( *p* = 0.1547).

A subgroup analysis was conducted to examine changes in SDS scores between participants with and without baseline depression. Although the mean score reduction appeared slightly greater in participants with depression (–3.33 ± 14.6) than in those without (–1.98 ± 7.5), the difference was not statistically significant ( *p* = 0.402). These findings suggest that CPAP therapy may improve symptoms regardless of baseline depression status.

This mean reduction of 4 points corresponds to an SD of approximately 0.4 to 0.5, which lies within the range often cited as the MCID for patient-reported outcomes. Notably, improvement in the cognitive and behavioral domain reached an SD of around 0.34, also within the MCID range.

The Wilcoxon signed-rank test confirmed these improvements, showing a significant difference in paired SDS scores (Z = –3.827; *p* < 0.001). Scatter plot analysis demonstrated a downward shift in posttreatment scores, with the regression line deviating below the 1:1 line, indicating overall improvement.

A subgroup analysis further revealed that SDS score reduction was slightly greater in participants with baseline depression (–3.33 ± 14.6) than in those without (–1.98 ± 7.5), though the difference was not statistically significant ( *p* = 0.402). This suggests that CPAP therapy may alleviate depressive symptoms regardless of their baseline status.

Furthermore, a positive correlation was observed between the pre- and posttreatment SDS scores: participants with higher baseline scores tended to show greater improvements (larger negative differences), suggesting that the degree of improvement was proportional to the severity of initial symptoms.

The SDS questionnaire was divided into specific components. No significant changes were seen in the “depressive mood” or “physical symptoms” components after CPAP therapy in either sex. However, significant changes were seen in the “cognitive and behavioral changes” component in the overall group ( *p* = 0.0010), specifically in males ( *p* = 0.0022), but not in females. Scores in the “functional impairment” decreased for both sexes, although this was not statistically significant. These findings indicate that long-term CPAP therapy reduces depressive symptoms in patients with OSA, especially in cognitive and behavioral domains, with more pronounced effects in the overall cohort and male participants, especially with prolonged use.

Subscale analysis using the SDS revealed that long-term CPAP therapy significantly reduced depressive mood and cognitive/behavioral symptoms, particularly among male participants. Although physical symptoms did not show significant improvement, functional impairment scores decreased in the overall sample and among participants. These findings suggest that CPAP therapy may have a positive impact on specific dimensions of depressive symptoms.

### Changes in SDS Subscale Scores Before and After CPAP Therapy

The SDS was used to evaluate the specific aspects of depressive symptoms affected by CPAP therapy. Significant reductions were observed in the overall group ( *p* < 0.001), as well as males ( *p* = 0.004) and females ( *p* = 0.026). Regarding physical symptoms, no significant change was observed in any group. Moreover, for cognitive and behavioral changes, significant improvements were observed in the overall cohort ( *p* < 0.001) and in males ( *p* < 0.001), but not in females ( *p* = 0.058). Finally, regarding functional impairment, a significant reduction was observed in the overall cohort ( *p* = 0.021) and in females ( *p* = 0.022), but not in males ( *p* = 0.125).

In exploratory subscale analyses ( Table 3 ), females showed reductions in depressive mood and functional impairment. However, given the limited female sample and multiplicity of tests, these findings are presented as exploratory and should be interpreted with caution.

**Table 3:** Changes in SDS subscale scores before and after CPAP

Subscale	Group	Pre-CPAP	Post-CPAP	*p* -value
Depressive mood (max 16)	Overall	6.79 ± 2.23	6.36 ± 2.05	< 0.001***
Physical symptoms (max 20)	Overall	7.63 ± 2.20	7.46 ± 2.03	0.588
Cognitive & behavioral changes (max 20)	Overall	8.00 ± 2.44	7.18 ± 1.95	< 0.001***
Functional impairment (max 28)	Overall	16.73 ± 4.61	16.05 ± 4.19	0.02 *

**Abbreviations:** CPAP, continuous positive airway pressure; SDS, Self-Rating Depression Scale.

**Notes:** Values are expressed as mean ± standard deviation. The *p-* values are based on paired t-tests, with significance levels of *< 0.05,**< 0.01, and ***< 0.001.

Furthermore, we examined the association between CPAP treatment duration and changes in SDS scores. No significant correlations were observed between treatment duration and either pre- or posttreatment SDS scores, nor with the difference between them.

In an additional analysis exploring the factors contributing to changes in the SDS scores, we observed that apart from age and the proportion of REM sleep (total sleep time), no other variables showed significant associations. Cluster analysis did not reveal any clear patterns linking SDS improvement to distinct clinical phenotypes. Furthermore, binary logistic regression using the SDS score change (above vs. below the median) as the dependent variable demonstrated no significant odds ratios for any polysomnography parameters or comorbidities. These results suggest that, within the scope of this dataset, no single factor could be identified as a strong predictor of SDS improvement following CPAP therapy ( Supplementary Table S1 ).

We attempted to detect dominant clusters using cluster classification but were unable to identify specific groups ( Supplementary Table S1 ).

Additionally, when comparing the group with good (≥ 70% for ≥ 4 h) and poor adherence, the former showed a higher improvement rate in SDS (particularly in terms of depressive mood and changes in cognition and behavior), as shown in the Supplementary Table S1 .

## Discussion

This study suggested that long-term CPAP therapy reduced depressive symptoms in patients with OSA, with exploratory analyses indicating more pronounced effects among males in domains related to cognition and behavior, such as fatigue, motivation, and decision-making. However, as this was an observational pre–post study without a control group, these findings should be interpreted as associations rather than proof of causality. Alternative explanations such as natural recovery, regression to the mean, or expectancy effects may also have contributed to the observed improvements. In patients using CPAP for ≥ 2 years, SDS scores improved significantly overall (40.2–37.5, *p* = 0.0474) and in males (38.3–35.4, *p* = 0.0490), with a nonsignificant trend in females (42.4–38.5, *p* = 0.1547).

These findings concur with those of earlier meta-analyses, demonstrating the efficacy of CPAP in alleviating depressive symptoms in patients with OSA. 6 7 We also extended the evidence base with ≥ 2 years of follow-up data. Our findings corroborate earlier reports that females may derive less psychological benefit from CPAP therapy, possibly due to sociocultural or hormonal factors, as well as challenges in adherence. 25 26

The decrease in SDS scores is consistent with the hypothesis that effective treatment of the physical symptoms of OSA can positively impact mental health. 10 12 These findings align with the notion that sleep quality and psychological well-being are interconnected. 11 12 13 14 In patients using CPAP for ≥ 2 years, improvements in SDS scores were observed.

The prevalence of depression in our study was 9.4%, which falls within the range reported in previous studies on patients with OSA (typically 10–20%). This suggests that our sample reflects a representative level of the depressive symptom burden in this population. Although the prevalence aligns with prior findings, its direct correlation with SDS score changes was not demonstrated in our study, indicating that other factors may have contributed to the observed improvement.

Additionally, these improvements could partly reflect nonspecific effects rather than CPAP therapy alone. Alternative explanations, such as natural recovery over time, regression to the mean with repeated assessments, or expectancy/placebo effects cannot be excluded. Given the absence of a control group, these possibilities must be acknowledged when interpreting our results. Moreover, although the overall change in SDS was statistically significant but modest, contextualization using MCID benchmarks (SD: 0.3–0.5) suggests that the observed 4-point reduction (SD: ∼ 0.4–0.5) may be clinically meaningful. The cognitive and behavioral subdomain, which showed an improved SD of approximately 0.34, also falls within this range, indicating potential relevance for daily functioning. Improvements in SDS scores appear to be primarily driven by changes in cognitive and behavioral aspects such as fatigue, irritability, motivation, and decision-making abilities. 11 12 13 14 15 20

This study contributes to the growing body of literature on the multifaceted benefits of CPAP therapy. Previous studies have focused primarily on the physical health outcomes associated with this treatment, such as improved cardiovascular health and reduced daytime sleepiness. 16 17 21 22 Our findings extend this understanding by highlighting the potential psychological benefits and offering a more comprehensive view of patients' well-being.

Comparing these results with existing research, our study underscores the importance of considering physical and psychological aspects in the treatment of OSA. 18 19 27 28 The significant reduction in depressive symptoms suggests that clinicians should consider the psychological benefits when prescribing CPAP therapy for this cohort. 19 28 Ensuring treatment adherence at the conventional threshold (≥ 4 h/night on ≥ 70% of nights) could improve physical health outcomes and enhance the overall quality of life by reducing depressive symptoms. This study underscores the need for healthcare professionals to monitor and encourage effective use of CPAP, considering its broader impact on patients' lives. 17 20

Our data showed that females tended to have higher baseline SDS scores but, given the small number of female participants, these sex-specific responses should be regarded as exploratory and interpreted with caution. This disparity may reflect differences in OSA manifestations, mood disturbances, neurobehavioral performance, and fatigue, as measured by the Profile of Mood States. Contrary to expectations, CPAP did not significantly improve the health-related quality of life in females compared to males, perhaps due to greater sensitivity to sleep disturbances and adaptation. Additionally, sociocultural and relational factors, such as marital status, may affect CPAP adherence in females. These findings underscore the need for a nuanced understanding of the sex-specific CPAP responses in OSA.

Beyond our cohort, there is growing global interest in female OSA, as they often present with diverse and sometimes atypical symptoms, which may be related to anatomical and hormonal differences. Progesterone and estrogen stimulate ventilation and protect against upper airway collapse. Additionally, women show greater sensitivity to blood gas changes, lower arousal thresholds, faster ventilatory stabilization, and lower loop gain compared with men. Furthermore, female patients frequently carry a higher psychosocial burden, which may influence symptom perception and treatment response. 29 The American Academy of Sleep Medicine 30 has also launched initiatives focusing on women's sleep health. These developments highlight the importance of establishing tailored sleep care strategies for female patients with OSA in future clinical practice.

From a clinical perspective, CPAP may contribute to the physical management of OSA and psychological recovery. The improvements observed in the cognitive and behavioral subdomains of the SDS indicate that it can enhance daily functioning beyond mood regulation. Better adherence was associated with greater improvement, reinforcing the importance of encouraging long-term use.

This study has certain limitations. First, the absence of a control or comparison group, such as patients with OSA who did not receive CPAP therapy or alternative treatments, precludes attributing the improvements in depressive symptoms definitively to CPAP therapy. Therefore, the observed changes should be interpreted with caution, as the design does not allow us to exclude nonspecific effects such as natural recovery or placebo responses. Other factors, including natural recovery, lifestyle changes, and placebo effects, may have contributed to this finding.

Second, qualitative factors influencing CPAP adherence (e.g., mask discomfort or perceived treatment burden) were not assessed. Furthermore, the relatively small number of female participants may have reduced the ability to detect sex differences. Finally, although symptom improvement may suggest a neuropsychological mechanism, no physiological parameters (e.g., functional magnetic resonance imaging or biomarkers) were evaluated.

Future studies should incorporate randomized controlled trials or matched control groups to enhance the causal inferences regarding the psychological effects of long-term CPAP therapy. Unintentional leak data, although obtainable, were not standardized across devices (different reporting units and formats) and, thus, were not analyzed.

Another important limitation is that depressive symptoms were assessed exclusively with the SDS, a self-reported instrument. Although the SDS is validated and widely used in Japan, self-report measures are subject to recall bias, social desirability bias, and reduced sensitivity particularly at mild-to-moderate severity levels. Future studies should therefore incorporate complementary clinician-rated scales (e.g., Hamilton Depression Rating Scale) and objective biomarkers (e.g., neuroimaging or inflammatory markers) to corroborate and extend the present findings.

### Future Directions

Further randomized controlled trials are required to establish causality and explore the underlying mechanisms through brain imaging and neurochemical analyses. Studies comparing CPAP with antidepressants or combination therapies may help tailor interventions, especially for patients with comorbid depression. Moreover, it is important to investigate how improvements in depressive symptoms translate into enhanced quality of life in patients with OSA.

Although our study provides valuable insights into the association between long-term CPAP therapy and depressive symptoms in patients with OSA, it has certain limitations. As previously noted, the pre–post within-subjects design and lack of a control group limit causal inference and attribution of effects solely to CPAP therapy. Furthermore, the relatively small sample size, especially in subgroups such as patients with comorbid depression, limits the generalizability of our findings. Additionally, the possibility of selection bias should be considered, as the participants were recruited from a single clinical center. The use of self-reported measures, such as the SDS, introduces the risk of measurement bias.

Moreover, the variability in CPAP adherence and individual patient responses suggests the need for personalized treatment approaches. Future studies should use longitudinal or randomized controlled designs to explore the potential mechanisms linking OSA, CPAP therapy, and depressive symptoms, including sleep quality, hypoxia, and systemic inflammation. Comparisons with alternative treatments, such as surgical interventions, are also warranted to provide a broader therapeutic perspective. 16 21

## Conclusion

Our study provides valuable insights into the relationship between long-term CPAP therapy and depressive symptoms in patients with OSA. Our findings suggest that CPAP therapy, which is primarily known for its effectiveness in treating the physical symptoms of OSA, significantly contributes to the reduction of depressive symptoms. This underscores the importance of a holistic treatment approach that considers psychological and physical health outcomes. Although the results are encouraging, the pre–post within-subjects design requires cautious interpretation. Further longitudinal and interventional studies are necessary to better understand the causal links and underlying mechanisms between CPAP therapy and mental health improvement. This study advocates an integrated treatment strategy for OSA, highlighting the need to address the physiological challenges and the mental wellbeing of patients.
