# Supplementary material for: Alleviating Depressive Symptoms Through Continuous Positive Airway Pressure Therapy: Long-Term Effects on Patients with Obstructive Sleep Apnea
Source: Sleep Sci. 2026 Nov 6;19(3):1–9. doi: 10.1055/s-0046-1819597 (PMC13743270; doi:10.1055/s-0046-1819597)
Supplement: Supplementary file 1 [file 10-1055-s-0046-1819597-s250432.pdf]

Table 1 Changes in SDS Subscale Scores Before and After CPAP, Stratified by Sex

Subscale	Group	Pre-CPAP	Post-CPAP	*p*-value
Depressive Mood (Max 16)	Overall	6.79 ± 2.23	6.36 ± 2.05	< 0.001 ***
	Male	6.69 ± 2.16	6.32 ± 2.04	0.004 **
	Female	7.31 ± 2.56	6.58 ± 2.16	0.026 *
Physical Symptoms (Max 20)	Overall	7.63 ± 2.20	7.46 ± 2.03	0.588
	Male	7.55 ± 2.18	7.38 ± 2.08	0.567
	Female	8.00 ± 2.32	7.85 ± 1.74	0.965
Cognitive & Behavioral Changes (Max 20)	Overall	8.00 ± 2.44	7.18 ± 1.95	< 0.001 ***
	Male	7.95 ± 2.41	7.13 ± 1.89	< 0.001 ***
	Female	8.27 ± 2.65	7.42 ± 2.25	0.058
Functional Impairment (Max 28)	Overall	16.73 ± 4.61	16.05 ± 4.19	0.021 *
	Male	16.34 ± 4.30	15.92 ± 4.13	0.125
	Female	18.77 ± 5.65	16.73 ± 4.51	0.022 *

Abbreviations: CPAP, continuous positive airway pressure; SDS, Self-Rating Depression Scale.
Values are expressed as mean ± standard deviation.
P-values are based on paired t-tests.
Significance level: $p < 0.05$ (*), $p < 0.01$ (**), $p < 0.001$ (***).

 © 2026. The Author(s).
